# Barriers and facilitators of employment in severe mental illness: an umbrella review

**DOI:** 10.3389/fresc.2025.1731096

**Published:** 2026-01-22

**Authors:** Ana Maria Radu, Balazs Feher-Gavra, Andrei Rusu

**Affiliations:** 1 Department of Psychology, West University of Timișoara, Timișoara, Romania; 2 Scientific Collective for Research in Neuroscience, Clinical Psychiatric Hospital “Dr. Gh. Preda”, Sibiu, Romania; 3 Institute for Advanced Environmental Studies (ICAM), West University of Timișoara, Timișoara, Romania

**Keywords:** employment, occupational rehabilitation, psychiatric diagnosis, severe mental illness, systematic review

## Abstract

**Introduction:**

Employment is one of the most important means of meeting an individual's psychological, social, and economic needs. Severe mental health illness (SMI) poses a significant challenge to managing psychological resources, which can further impede one's employability. The purpose of this research is to investigate the contextual factors associated with employment for individuals suffering from SMI, for the benefit of both their well-being, as well as the wider economic and social context they live in.

**Methods:**

This umbrella review evaluates the available literature from PubMed and PsycINFO. It included systematic reviews, meta-analyses, and scoping reviews, for which a quality appraisal was conducted using the AMSTAR 2 Checklist and the Scoping Review Checklist.

**Results:**

Our search identified 40 reviews, from which we pulled seven evidence-based factors associated with employment, four facilitators, and three barriers. Receiving individual placement and support represents the facilitator that was most extensively researched, with reported employment rates up to 61%, while negative symptoms represent the most robustly identified barrier.

**Discussion:**

Overall, the synthesis of evidence highlights underlying factors that might shape employment for individuals with SMI and identifies interventions that hold potential for further development. It also underscores the necessity for conceptual consistency and reveals significant heterogeneity in the literature, which diminishes the generalizability of the results.

**Systematic Review Registration:**

https://www.crd.york.ac.uk/PROSPERO/view/CRD42023460410, PROSPERO CRD42023460410.

## 1 Introduction

Employment of people with severe mental illness (SMI) posits psychological, social, and economic benefits for both the individual and society. On a personal level, employment can contribute to symptom management and reduction, increase self-esteem, quality of life, and self-sufficiency (1, 2). It was also associated with a lower risk of substance abuse and psychological distress (3). At a group level, it provides people with SMI an opportunity to contribute to the society they live in, foster inclusion, and reduce stigma, which in turn can lower health care costs and disability pensions (1). There is significant research [e.g., (3–5)] that focuses on understanding and facilitating the employment of people with SMI, and yet, their employment rates remain significantly low. Therefore, it is essential to identify and examine the factors associated with employment in order to develop an integrated overview.

Employment can shape individual well-being, which can foster autonomy while also aiding in recovery from various health challenges (2). Empirical research underscores these advantages. A systematic review by Modini et al. (3) synthesized evidence from multiple high-quality studies, showing that gainful employment not only enhances overall life satisfaction but also mitigates mental health vulnerabilities. Specifically, their analysis revealed that employment is associated with reductions in anxiety and depressive symptoms, whereas unemployment increases the risk of substance abuse and broader psychological distress. The effect sizes indicated moderate to strong protective benefits of work, particularly when paired with supportive supervision and high-quality job conditions. Similarly, Üçok et al. (6) found that employment correlates with reduced psychopathology and greater psychosocial integration. Nevertheless, certain diagnostic subgroups face more challenges in obtaining or maintaining employment. For example, individuals with schizophrenia report markedly lower employment rates (10%) compared with those with bipolar disorder (34%) (4). In general, the greater the severity of psychiatric illness, the lower the likelihood of employment or higher income (7).

Moreover, substantial evidence supports the positive impact of employment and supported employment models, which can be highly effective in enhancing self-esteem, fostering self-sufficiency, and improving overall quality of life. At the same time, they enable individuals to contribute to their communities and provide stability in the family context through greater independence and capability (1). This perspective is supported by various studies, such as Pinho et al. (8), who conducted a cross-sectional study of outpatients with schizophrenia, and revealed that employed participants or those studying, living autonomously, or with a family member, had significantly higher quality of life scores, with social support satisfaction as a mediator. Further, Jäckel et al. (5) found statistically significant associations between sustained competitive employment and reduced psychiatric hospitalizations, reporting an average reduction of 45% over 18 months. In contrast, Prause et al. (9) observed only marginal effects, with employment status explaining less than 5% of the variance in quality of life among psychiatric outpatients. This suggests that contextual factors such as job quality and social support may moderate the impact of employment on mental health outcomes. Similarly, Van Dongen (10) found no statistically significant associations between employment status and either quality of life or self-esteem, suggesting that intrinsic factors such as symptom management may outweigh vocational influences. Moreover, Agerbo (11) provided contradictory evidence regarding employment among people with SMI, showing that work paradoxically increased suicide risk. This effect was particularly pronounced in certain occupations, such as physicians, and diminished after controlling for income and marital status, potentially reflecting workplace stressors or unmet support needs. Taken together, these findings suggest that while employment can provide meaningful psychological, social, and clinical benefits for individuals with SMI, its effects are not universally positive and may be shaped by contextual and individual factors such as job quality, social support, and occupational stressors. This mixed evidence underscores the need for structured, evidence-based interventions that maximize the benefits of employment while minimizing potential risks, ensuring that work functions as a protective rather than detrimental factor in the recovery process.

Various evidence-based interventions have been developed to enhance employability among individuals with SMI, with Individual Placement and Support (IPS), a structured supported employment (SE) model, being the most extensively researched. A recent umbrella review (12), synthesized 26 reviews, focusing on mostly on IPS and its modified forms, revealing that while standard SE yields positive employment outcomes in 60% of cases, augmented SE, integrating interventions like cognitive remediation and social skills training, achieve higher employment rates (71% positive outcomes), improved quality of life, and reduced psychiatric hospitalizations. The review also draws attention to the importance of evaluating a broader area of interventions and approaches to enhance employment and recovery of people with SMI.

Consequently, the current umbrella review aims to comprehensively overview the psychosocial factors associated with employment among individuals with SMI or other psychiatric diagnoses. It synthesizes evidence from systematic reviews, meta-analyses, and scoping reviews to address the following questions: What are the psychological factors associated with employment status for individuals with severe mental health issues? What are the positive correlates of employment and the negative ones? Are there variations in these relationships based on the type of diagnosis? Are there variations in these relationships based on the education level/social functioning? Do previous hospital commitments act as hinderers or moderators for gaining employment for patients with a psychiatric diagnosis? Does enrollment in different rehabilitation programs act as facilitator for gaining employment for patients with a psychiatric diagnosis? Hence, by compiling evidence on psychological, clinical, and social determinants of employment, this review seeks to inform tailored strategies that promote well-being through vocational integration.

## 2 Materials and methods

The present study is an umbrella review, synthesizing evidence from systematic reviews, meta-analyses, and scoping reviews with the purpose of providing a broad overview of empirical findings, integrating data across sources, and identifying gaps that require further investigation. Consistent with Cochrane guidance, umbrella reviews use clear and systematic methods to find and assess multiple systematic reviews on related topics. This method is useful both for summarizing the existing synthesized evidence and for answering broader questions that individual systematic reviews may not have covered. By following predefined protocols, comprehensive search strategies, and transparent reporting standards, umbrella reviews help reduce bias and improve the reliability of the findings (13). Transparent reviews are essential for identifying knowledge gaps, developing theory, and informing policymakers (14). This review was registered on PROSPERO (CRD42023460410) and follows the Preferred Reporting Items for Overviews of Reviews (PRIOR; see Supplementary Material 1).

### 2.1 Search strategy

The initial literature search was conducted on May 19, 2023, covering publications from PubMed and PsycINFO, and was complemented by a hand search of the reference lists of the included reviews. Search terms combined descriptors of “severe mental illness”, “employment”, and “reviews” (e.g., “psychiatric”, “mental disorder”, “vocational rehabilitation”, “systematic review”, “scoping review”). The complete search strategy is provided in Supplementary Material 2. Searches were restricted to articles in English and Romanian, as these are the languages mastered by the research team. However, we did not find any studies available only in Romanian; therefore, all included studies were in English. An updated search was conducted in August 2025 to cover the 2023–2025 period, with reference lists of newly identified studies also hand-searched.

### 2.2 Study selection

Study selection was conducted independently by two researchers, with inclusion confirmed against pre-specified criteria. Eligible studies were included based on the following criteria:

#### 2.2.1 Population

Adults with a psychiatric diagnosis, given before the inclusion in the study by a professional or established under DSM criteria of diagnosis, individuals with SMI who meet the DSM 4 or 5 criteria of diagnosis, individuals with SMI identified based on screening with validated measures and cut-off criteria (e.g., Beck Depression Inventory-II), and persons with psychiatric disability. Only populations of legal working age were considered.

#### 2.2.2 Intervention

Any intervention designed for individuals with SMI that aimed to improve employment outcomes was considered eligible. Occupational interventions for people with a psychiatric diagnosis or SMI were defined as programs that support individuals in gaining, sustaining, or regaining employment. Examples include prevocational training, supported employment, vocational services, individual placement and support, as well as psychological, educational, and social interventions such as cognitive remediation and social skills training.

#### 2.2.3 Psychosocial factors

Psychological factors associated with employment that could influence the process of gaining, maintaining, or returning to work for people with SMI. Both positive factors (facilitators) and negative factors (barriers) were considered. Social and clinical factors could be intertwined with psychological factors as they can directly impact one's psychological state of mind, which could further influence employment status. Examples of such factors are diagnosis severity, type of diagnosis, hospitalization period, stigma, comorbid health conditions, neurocognitive functioning, social functioning, side effects of medication, enrolment in a type of occupational, vocational, or psychiatric rehabilitation program, marital status, age, employment history, or community functioning.

#### 2.2.4 Outcomes

Only reviews that focused on, or also on, employment as an outcome were considered. Competitive employment, supported employment, and sheltered employment were accepted as outcomes. Vocational outcomes, such as internships or any other form of unpaid work, were not accepted as occupational outcomes. Also, studies focused on the impact of disability status for individuals with psychiatric diagnoses or severe mental health issues were excluded. The main reasons were the legislative discrepancies between countries concerning the circumstances in which the disability status was achieved or maintained.

#### 2.2.5 Types of studies

This review considered peer-reviewed systematic reviews, meta-analyses, and scoping reviews of observational studies (e.g., cohort studies, cross-sectional studies, case-control studies), longitudinal studies (e.g., cohort studies), and clinical trials (randomized/non-randomized). Reviews of qualitative studies or non-systematic reviews (e.g., narrative) were excluded.

### 2.3 Screening and data extraction

All citations were exported from the databases in Rayyan (15), where duplicates were removed. All potential studies were reviewed by two researchers (AMR and BFG). The first study selection phase consisted of screening the titles and abstracts. The resultant studies were subjected to full-text screening, and any discrepancies were resolved by reaching consensus through discussions. If consensus on eligibility could not be reached, a third researcher (AR) was asked as a tiebreaker. The extracted information consisted of general data about the article (author, title, study design, no. of participants, study limitations), socio-demographic characteristics of participants (type of population, age, gender, marital status), and clinical information regarding diagnosis and co-morbid conditions. Data about the type of intervention used, its efficacy, impact on the employment status of participants, and employment duration were also extracted. Another category included in the data extraction form regarded the associated factors to employment (potential and evidence-based factors for gaining, keeping, and/or regaining employment, and/or barriers to employment).

### 2.4 Risk of bias assessment

Risk of bias was assessed using the AMSTAR-2 checklist (16) for the included systematic reviews and systematic reviews with meta-analysis. We chose the AMSTAR-2 checklist, following the recommendations of Fusar-Poli and Radua (17) for conducting umbrella reviews, because it was designed to assess systematic reviews that include both randomized and non-randomized studies in healthcare (16). The extracted data for the risk of bias assessment consisted of review method, search strategy, study selection, data extraction, data heterogeneity, use of statistical analysis, risk of bias assessment, funding, and declaration of competing interests. For the included scoping reviews, an evidence-based checklist following the framework proposed by Arksey and O'Malley (18) for improving scoping review quality, developed by Cooper et al. (19), was used. We only considered 20 items out of 22, as the last two items were optional and concerned stakeholders. The scale did not define a mean for the cumulative score of the reviews, so that reviews were ranked as high or low quality. It was designed as a critical appraisal instrument, and the more items are checked, the better the quality of the review.

### 2.5 Generative AI and research ethics

ChatGPT version 5 was used, given a single prompt, to generate a heatmap for illustrating the scoping review quality assessment results, after providing the input data in editable table format [following the criteria from The Scoping review Checklist; (19)]. On one hand, using AI-generated figures for data visualisation support, such as a heatmap for the quality appraisal of reviews in our article, brings a high-quality illustration in an efficient way. AI-generated figures represent an efficient, accessible, high-quality method for visual representations in an academic article. On the other hand, the AI-generated images could incorporate incorrect or fabricated details, therefore researchers should thoroughly verify the AI-generated figures and compare them with the raw data from their research. For a detailed discussion regarding the risks of AI usage for generating figures, see Skulmowski and Engel-Hermann (20). The raw date used in this case is openly provided in Supplementary Material 3.

## 3 Results

### 3.1 Study selection

**Figure 1:**
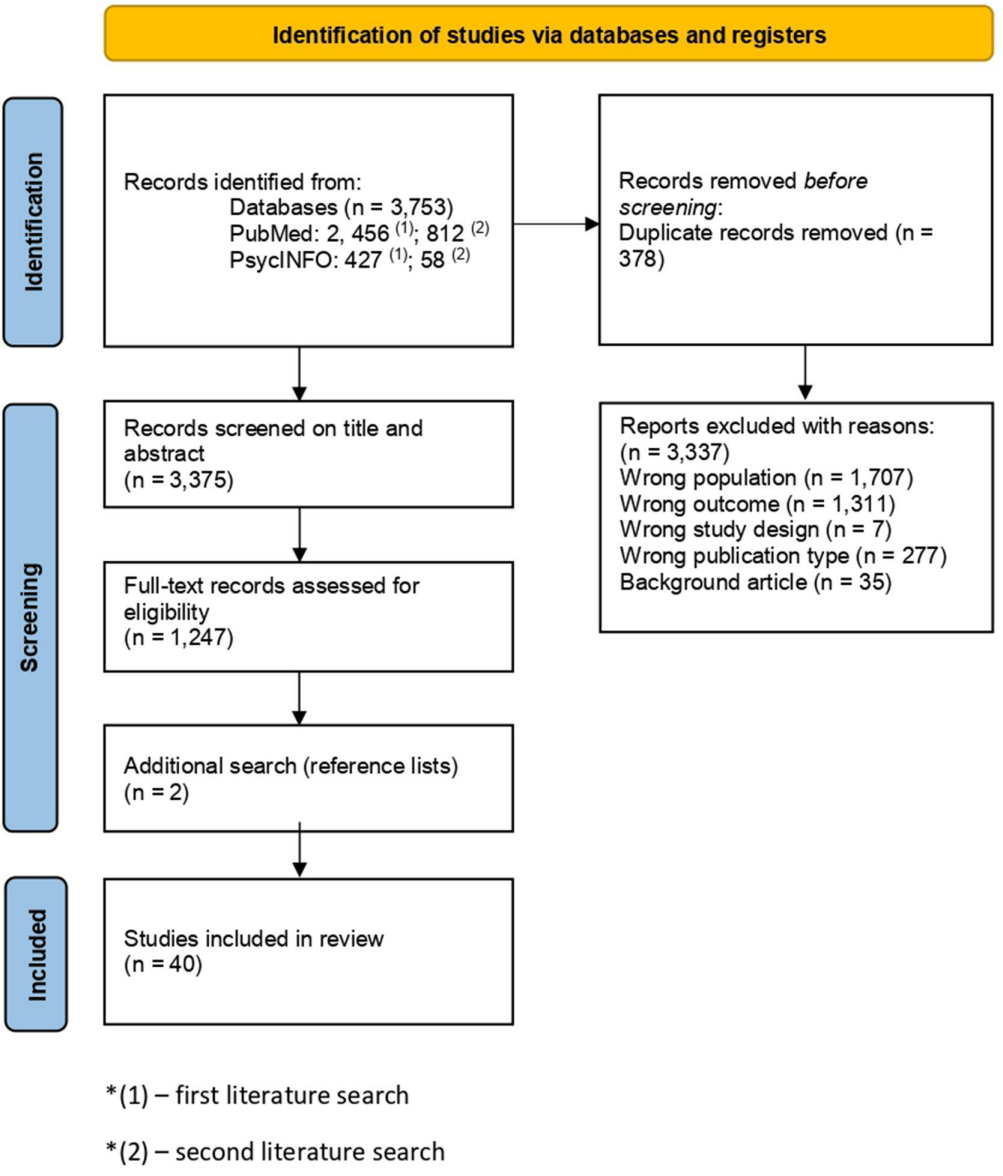
PRISMA flow chart of the literature, inclusion and exclusion (62). *(1) – first literature search. *(2) – second literature search.

The literature search pooled 3,753 articles from PsycINFO and PubMed, out of which 1,239 articles were pooled from the second literature search (covering the 2023–2025 period). The Rayyan online tool was used to record decisions. Hence, 3,375 articles remained after resolving duplicates from both searches. The abstract screening was conducted by two researchers following the inclusion criteria. Overall, 1,247 articles remained for full-text screening. After the researchers achieved consensus on which studies to include, 33 reviews were eligible for the umbrella review – initial search from inception to 2023. Five more reviews were identified by manually searching the reference lists of included studies, and two more reviews were added in the second literature search, resulting in a final sample of 40 included records (Figure 1). A complete reference list of excluded studies, together with the reasons for exclusion, is available in Supplementary Material 3.

### 3.2 Study characteristics

Among the 40 included reviews were 23 systematic reviews, 12 systematic reviews with meta-analysis, and five scoping reviews, which compiled 1062 studies. Reviews were published between 1998 (the earliest identified one) and 2025. The number of participants varied from 614 to 25,399. Eight reviews did not mention the total count of participants. The mean age varied between 15 and 65 years old; most of the participants were males and unmarried. However, 18 studies did not report the mean age, and only 10 reported the gender of the participants, and three studies reported the marital status. Many studies (*n* = 27) included samples from the schizophrenia spectrum or psychotic disorders, while six studies did not mention any specific diagnosis. Other reviews included samples of people with mood disorders, major depressive disorder, stress disorders, personality disorders, and autism spectrum disorder. Few studies reported comorbidities (*n* = 19); among the mentioned ones are substance use disorder, cognitive deficits, autism spectrum disorder, intellectual disability, depression, psychotic features, phobia, chronic pain, and mania. The employment operationalization, which was reported in 19 reviews, was similar between studies including aspects such as working for payment, being involved in an environment that provides a salary, or sustaining a paid activity. Most of the reviews (*n* = 34) included an intervention that considered gaining, maintaining, or returning to employment. Most interventions (*n* = 22) investigated the efficacy of programs of supported employment or variations of this program, such as Individual Placement Support (IPS). Extracted data regarding included studies about study design and participant characteristics are described in Tables 1, 2.

**Table 1:** SE interventions and their efficacy for employment outcomes.

Article	Population	No. of studies	Objective	Interventions	Intervention efficacy
Abidin et al. (52)	Schizophrenia and other SMI	24–9 studies on SE or ISE	Examine the effectiveness of different interventions on both vocational and non-vocational outcomes.	SE, Integrated supported employment (ISE)	SE - 4 studies reported employment outcomes and compared efficiency to other vocational services, the employment rates varied between 42,5% for the SE group and 70% compared with 11% and 31% for control group; ISE - 5 studies reported employment outcomes and compared them with conventional vocational services, the employment rates varied between 63% and 82,8% for ISE compared with 33,3% and 61,5% for control., job tenure was higher for ISE groups varying between 23,84 weeks to 49,94 compared with 9,91 weeks to 36,17 weeks. No efficiency improvement was reported when ISE was combined with cognitive remediation.
Bond et al. (29)	People with SMI	15	To examine the effectiveness of IPS in both US and non-US studies.	IPS	55,7% of the IPS group obtained employment compared with 22,6% of the control group, the effects were larger for US studies (0.84) compared with non-US studies (0.67); 8 studies reported employment retention and IPS participants worked twice as many weeks per year compared with control.
Bond et al. (30)	Young adults with early psychosis	28	To examine the effectiveness of IPS for young adults with SMI.	SE	The follow-up employment rate was 49% among supported employment participants, compared to 29% in the control group, χ^2^ (1) = 21.6, *p* < 0.0001, with an effect size of 0.41. After adjusting for baseline employment levels, the increase from admission to follow-up was 41% in the supported employment group vs. 17% in controls, corresponding to an effect size of 0.54.
Bond et al. (31)	First-episode psychosis and other SMI	7	To examine the effectiveness of IPS across RTCs.	IPS	The employment rates varied across studies from 33% to 82% for IPS groups compared with 5% to 42% for control, at 6 months follow up the number of worked weeks varied for IPS group between 148.2 and 8.63 compared with 97.8 and 7 days for control, in 6 studies the employment rate at follow-up was at least 20% greater for IPS than for the control group.
Brinchmann et al. (32)	Adults with mental illness	27	Examines IPS efficacy across different countries.	IPS	The overall meta-analysis indicates that individuals receiving IPS were over twice as likely to secure competitive employment compared to those receiving TAU [RR = 2.07, 95% CI (1.82–2.35), *p* < 0.0001]). The efficiency was moderated by legal protection, but the effect was marginal. Results can be generalized.
Campbell et al. (33)	People with psychotic disorder, mood disorder or other SMI	4	To examine which sub-groups benefit more for IPS.	IPS	The results reported strong effects for obtaining employment through IPS (0.96), for total weeks worked (0.79), and maintaining employment (0.74). Across studies, stronger effects were reported for psychotic disorders compared with mood disorders.
Crowther et al. (34)	People with schizophrenia and other SMI	18	To evaluate the effectiveness of pre-vocational training and SE	SE	SE shows greater effectiveness at 24 months [RR = 0.92, 95% CI (0.85–0.99)] and 36 months [RR = 0.88, 95% CI (0.82–0.96)]. Evidence from five trials further indicates that supported employment is superior to prevocational training.
de Winter et al. (28)	People with schizophrenia, major depressive disorder, anxiety disorder, PTSD, SUD and other SMI	32–31 reported employment outcomes	To examine if the effectiveness of IPS differs across subgroups.	IPS	48.8% of IPS participants were employed at follow-up compared to 28.3% of controls [OR = 2.62, 95% CI (2.37–2.89), *p* < 0.01], with moderate heterogeneity (I^2^ = 74%). Effects were stronger for SMI compared with CMI [χ^2^ (1) = 10.79; *p* < 0.01], people with schizophrenia spectrum disorders are more likely to have positive employment outcomes [χ^2^ (1) = 18.24; *p* < 0.01], followed by individuals with MDD [χ^2^ (1) = 5.36; *p* < 0.05], 23 studies included job tenure and the effects were small compared to control group [*d* = 0.41 95% CI (0.30–0.52), *p* < 0.01] and moderately heterogeneous [I^2^ = 77% (69%–83%); *p* < 0.01].
Heffernan et al. (35)	People with psychotic illness	5	To investigate the effectiveness of IPS in the UK.	IPS	In one 18-month trial across European centers, 55% of participants in the IPS group worked at least one day compared with 28% in conventional vocational services, a difference of 26.9% [95% CI (16.4–37.4)]. A second trial found no significant difference at one year in achieving 30 days or more of competitive employment (13% IPS vs. 7% control). Two additional trials reported IPS placement rates of 41% and 39% at one year. IPS patients maintained jobs for 214 days compared to 108 days for traditional vocational service, but one study found no difference with the median hours worked per week or length of job tenure between the control and intervention group (median 13 weeks vs. 18 weeks, *p* = 0.23.)
Hellstrom et al. (27)	Adults with schizophrenia, bipolar disorder and depression, SUD or forensic psychiatric diagnoses	13	To evaluate the effectiveness of IPS across different diagnoses on return to competitive employment.	IPS	Across all studies, the IPS group were 1.92 more likely to be in competitive employment at any time during the 18-month follow-up compared to service as usual [95% CI (1.53–2.42)], strong effects were identified for schizophrenia [OR: 2.07 95%CI (1.58–2.73)] and bipolar disorder [OR: 2.37 95%CI (1.27–4.43)], while for depression the results were not significant. The IPS had better outcomes in terms of maintaining employment, 221,5 h compared to 116,8 h for service as usual. For schizophrenia and bipolar disorder, differences with similar magnitudes were reported (adj. EMDs: 109.1 h, 95% CI [60.5–157.7], 6.1 weeks, 95% CI [3.9–8.4], and adj. EMDs: of 108 h [95% CI (80.6–297.4)] and 6.7 weeks [95% CI (0.3–13.7)], and for depressive disorder no significant difference was observed compared to service as usual.
Killaspy et al. (36)	People with SMI	72–20 studies focused on SE and IPS	Evaluation of interventions for people with SMI to improve social and economic outcomes.	IPS ad SE	Employment rates for IPS or SE interventions varied between 32,9% and 79% compared to 34% up to 46,5% of participants who received service as usual. Three studies found no differences between groups.
Kinoshita et al. (37)	People with schizophrenia, MMD or bipolar disorder	14–11 studies reported outcomes on competitive employment	Compare the effectiveness of SE to other vocational rehabilitation interventions or TAU and evaluate factors that could influence effectiveness.	SE, IPS, Augmented SE	Only one study reported the number of days in competitive employment of two groups, SE and other vocational approaches [MD 70.63, 95% CI (43.22, 98.04)], and two studies reported days in any form of paid employment, with SE being superior to other vocational services [MD 84.94, 95%CI (51.99, 117.89)]. Job tenure for competitive employment had favorable outcomes for SE compared to vocational approaches [MD 9.86, 95% CI (5.36–14.36)], but no significant effect was found for any paid employment. A significant difference was reported between SE and other vocational services in 7 studies for gaining employment during the intervention [RR 3.24, 95% CI (2.17–4.82)].
Modini et al. (3)	People with SMI	19	Evaluate the effectiveness of IPS across different countries and economic contexts.	IPS	The pooled analysis showed that participants receiving IPS were 2.4 times more likely to obtain competitive employment compared with those in traditional vocational rehabilitation [95% CI (1.99–2.90)]. Moderate heterogeneity was observed across studies (Q = 62.70, I^2^ = 66.5%, *p* < 0.001). In the first year, IPS participants were 2.59 times more likely to gain competitive employment than those in traditional vocational rehab [95% CI (1.88–3.56)], and 2.41 times more likely in the second year [95% CI (1.96–2.97)]. IPS was more effective in regions with higher economic status [*β* = 0.13, 95% CI (0.00–0.25), *p* = 0.045].
Noyes et al. (38)	Adults with schizophrenia, bipolar disorder, MDD and other SMI	57	Evaluate interventions for people with SMI to improve and maintain education and employment.	IPS, ISE, Prevocational training, Clubhouse model, Occupational therapy (OT)	Employment rates favored the intervention in all comparisons: ISE 31% vs. Clubhouse 7%, IPS 73% vs. Clubhouse 18%, IPS 24.9% vs. conventional vocational rehabilitation 14.3%, and OT-led prevocational + CBT 71% vs. TAU 16%. At 6 months, IPS participants worked more weeks and days than controls, with comparable total hours, showing higher overall engagement.
Richter et al. (39)	People with psychosis and schizophrenia	28	To investigate the effectiveness of non-trial SE programs and to compare effectiveness to SE in RCTs.	IPS	Competitive employment rates were higher for IPS in both trial and non-trial (SE routine programs: 0.43 (95% CI [0.37–0.50]; SE programs in trials: 0.50 [0.43–0.56] compared with prevocational training; PVT routine programs: 0.17 [0.11–0.23]; PVT programs in trials: 0.22 [0.16–0.28]).
Rinaldi et al. (40)	Young people with first-episode psychosis	16	To evaluate if young people with first-episode psychosis want to work, what are their challenges, what are the most effective interventions and at what costs.	IPS	IPS was shown to increase competitive employment rates for people with longer-term mental health conditions compared to traditional vocational rehabilitation. In five IPS studies, the combined employment and education rate was 69% for IPS participants vs. 35% for controls.
Smith et al. (41)	People with ASD and other SMI	46 studies, 25 SMI, 5 ASD, 16 various disability	To investigate the effectiveness of interventions to increase employment outcomes for people with various disabilities.	IPS, SE, Supported education	Participants assigned to IPS were substantially more likely to engage in vocational activities, with a significantly higher proportion achieving at least some competitive employment (three studies involving individuals with severe mental illness). In a study of homeless young adults with psychiatric disorders, IPS participants were significantly more likely to work during the 10-month study period and accumulated more months of employment compared with those receiving standard care (one study). Two studies introduced CBT components to SE, but no significant differences were found. One study augmented SE with job simulations for people with ASD, which concluded that participants gain employment faster if they benefit from simulation.
Thompson et al. (42)	Young people with psychotic and mood disorders, schizophrenia or schizoaffective disorder, MDD and anxiety disorder	10	To evaluate interventions for educational and employment outcomes.	IPS, SE, Adapted IPS, vocational Support etc.	Five RCTs found significantly higher employment rates among participants receiving vocational interventions. Four RCTs of IPS interventions also examined total employment duration, with three reporting significantly more weeks worked in the IPS group compared with controls. Findings from three quasi-controlled IPS trials tailored for young people were consistent, showing superior employment outcomes for participants in the vocational intervention groups.
Twamley et al. (43)	People with schizophrenia	11–9 studies focused on IPS or SE	To evaluate vocational outcomes from RCTs	IPS	The employment rates for IPS or SE groups varied between 32% and 61% compared with 19% and 32% for control. Across five trials, 51% of participants receiving IPS or SE achieved competitive employment at any point during the study, compared with 18% of those receiving traditional vocational rehabilitation, corresponding to a weighted mean effect size of 0.79.

**Table 2:** Potential facilitators and barriers associated with employment for people with SMI.

Article	Potential factors associated with gaining employment	Factors associated with keeping employment	Barriers of employment
Arbesman et al. (49)	Cognitive support, daily living activities	x	x
Bond et al. (31)	Younger age (30 years and under)	x	x
Bouwmans et al. (54)	Awareness of illness, verbal memory, treatment as an outpatient, younger age, more education, male sex, availability of vocational services.	x	x
de Winter et al. (28)	x	x	Substance and alcohol use
Garrelfs et al. (58)			Psychiatric comorbidity
Gilbert et al. (56)	Educational attainment	x	Low education attainment, depression, cognitive impairment
Hellstrom et al. (27)	x	x	Depressive symptoms, stigma attributed to substance use disorder
Jonsdottir et al. (59)	x	x	Unemployment history
Marwaha et al. (55)	x	x	Negative symptoms
McKay et al. (51)	x	x	Jobs that were specifically set aside for persons with mental illness
Michon et al. (45)	More years spent in education, higher expectations of vocational future, less time between hospital and engagement in rehabilitation, less hospitalizations, higher interest in societal developments, higher scores on “aggressive syndrome”, more years spent in education, better prevocational rehabilitation work performance, being mentally ill for longer period, reception of rehabilitation subsidies, less negative symptoms, less conceptual disorganization, less disorder of relating, lower external locus of control, higher outcome expectations, less pronounced negative symptoms, less ‘depression-resigned’ coping, less impaired cognition, more fatalistic control beliefs, better social work performance, better social functioning	Better prevocational rehabilitation performance, less severe symptoms, being married, absence of criminal past, absence of psychiatric medication.	x
Noyes et al. (38)	x	x	Psychiatric symptoms, minimal or no previous work experience, low confidence and motivation, and long periods of being out of work, job markets, complex job search procedures, non- supportive working conditions, lack of meaningful work, and stigma associated with mental illness
Rinaldi et al. (40)	Having a good employment history within the last 5 years, motivation, self-esteem	x	Negative symptoms, stigma, discrimination, being unable to manage symptoms, the fear of disclosing diagnosis, the gaps in employment history, general functioning, depression, cognitive impairments
Sinclair et al. (24)		Stable housing, connectedness, collaborative and individualized care, peer and professional support, empowerment opportunities, and recovery-oriented organisational practices	x
Tsang et al. (57)	Younger age, marital status, higher social class, living in residential areas, cognitive functioning, ego strength, personal presentation, social skills, social contact, heterosexual relationship, family relationship, premorbid functioning, premorbid occupational performance, attitude toward work, work adjustment skills, availability of employment assistance, service in hospital, higher quality of life, male gender	x	Psychiatric symptomatology, neuropathology, severity of illness, use of neuroleptic drugs, age at first hospitalization, biological vulnerability, family psychiatric history, length of hospitalization, substance abuse, criminal record, relapse rate, previous hospitalizations, disability allowance
Twamley et al. (43)			Neuropsychological deficits, impairments in everyday functioning.

### 3.3 Quality appraisal

The quality assessment was completed with two instruments, AMSTAR 2 (16) for systematic reviews and meta-analyses, and the Scoping Review Checklist (19) for scoping reviews.

#### 3.3.1 Risk of bias in the included systematic reviews and meta-analyses

Out of 40 included reviews, 35 were appraised with AMSTAR 2 (16). According to the guidance from the checklist, there are 7 critical domains (protocol registration, adequacy of the literature search, justification for excluding individual studies, risk of bias, appropriateness of meta-analytical methods, consideration of risk of bias when interpreting the results of the review, assessment of presence of publication bias) without whom a review cannot be considered of “high quality” (16). Unfortunately, none of the included studies checked all the critical domains; therefore, none qualify as “high quality” nor even for “moderate”, as all of them have at least one critical weakness and were graded “critically low” or “low” (Table 3).

**Table 3:** Results of quality assessment for the systematic reviews (based on the AMSTAR2).

Article	Protocol registration	Adequacy of literature search	Justification for excluded studies	Risk of bias	Appropriateness of meta-analytical methods	Consideration of risk of bias	Publication bias	Global rating
Abidin et al. (52)	Yes	Yes	No	No	No meta-analysis	No	No meta-analysis	Critically low
Arbesman et al. (49)	No	Partial yes	No	No	No meta-analysis	No	No meta-analysis	Critically low
Battin et al. (50)	No	Partial yes	No	No	No meta-analysis	No	No meta-analysis	Critically low
Bond et al. (29)	No	Partial yes	Yes	No	No meta-analysis	No	No meta-analysis	Critically low
Bond et al. (30)	No	Partial yes	No	No	No meta-analysis	No	No meta-analysis	Critically low
Bond et al. (31)	Yes	Partial yes	No	No	Yes	No	No	Critically low
Bouwmans et al. (54)	No	Partial yes	No	No	No meta-analysis	No	No meta-analysis	Critically low
Brinchmann et al. (32)	No	Yes	Yes	Yes	Yes	Partial yes	Yes	Low
Campbell et al. (33)	No	Partial yes	No	No	Yes	No	No	Critically low
Chan et al. (46)	No	Yes	Yes	Yes	Yes	Partial yes	Yes	Low
Crowther et al. (34)	No	Partial yes	Yes	Yes	Yes	No	No	Critically low
de Winter et al. (28)	Yes	Partial yes	No	Yes	Yes	Yes	Yes	Low
Garrelfs et al. (58)	No	Partial yes	No	Yes	No meta-analysis	No	No meta-analysis	Critically low
Gilbert et al. (56)	No	Partial yes	No	No	No meta-analysis	No	No meta-analysis	Critically low
Heffernan et al. (35)	No	Partial yes	Yes	Yes	No meta-analysis	Yes	No meta-analysis	Low
Hellstrom et al. (27)	Yes	Partial yes	No	Yes	No meta-analysis	Yes	No meta-analysis	Low
Jonsdottir et al. (59)	No	Partial yes	No	No	No meta-analysis	No	No meta-analysis	Critically low
Killaspy et al. (36)	No	Partial yes	No	Yes	No meta-analysis	No	No meta-analysis	Critically low
Kinoshita et al. (37)	Yes	Partial yes	Yes	Yes	Yes	Yes	Yes	High
Kirsh et al. (48)	No	Yes	No	No	No meta-analysis	No	No meta-analysis	Critically low
Marwaha et al. (55)	No	Partial yes	No	No	No meta-analysis	No	No meta-analysis	Critically low
McKay et al. (51)	No	Yes	No	No	No meta-analysis	No	No meta-analysis	Critically low
Michon et al. (45)	No	Yes	Yes	No	No meta-analysis	No	No meta-analysis	Critically low
Modini et al. (3)	No	Yes	No	Yes	Yes	Yes	Yes	Critically low
Noyes et al. (38)	No	Partial yes	No	Yes	No meta-analysis	No	No meta-analysis	Critically low
Richter et al. (39)	No	Partial yes	No	No	Yes	No	Yes	Critically low
Rinaldi et al. (40)	No	Partial yes	No	No	No meta-analysis	No	No meta-analysis	Critically low
Sengupta et al. (44)	No	Partial yes	No	No	No meta-analysis	No	No meta-analysis	Critically low
Smith et al. (41)	No	Partial yes	No	Yes	No meta-analysis	No	No meta-analysis	Critically low
Thompson et al. (42)	No	Partial yes	No	No	No meta-analysis	No	No meta-analysis	Critically low
Tsang et al. (57)	No	Partial yes	No	No	No meta-analysis	No	No meta-analysis	Critically low
Tse et al. (53)	No	Partial yes	No	No	Yes	No	Yes	Critically low
Twamley et al. (43)	No	Partial yes	No	No	Yes	Yes	Yes	Critically low
van Duin et al. (47)	No	Partial yes	No	Yes	Yes	Yes	Yes	Critically low
Vinu & Georgiades (63)	Yes	Partial yes	No	Yes	No meta-analysis	Yes	No meta-analysis	Low

#### 3.3.2 Risk of bias in the included scoping reviews

**Figure 2:**
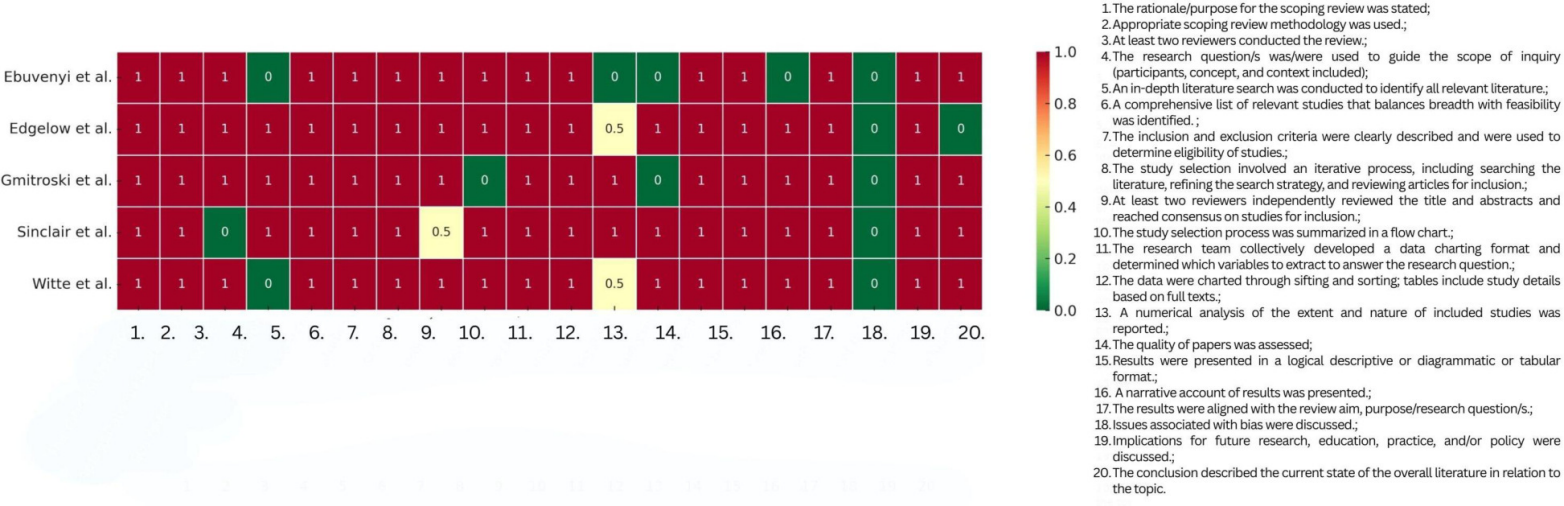
Heatmap of quality appraisal for Scoping Review Quality Assessment [based on Cooper et al. (19)].

Five scoping reviews were included in this umbrella review (21–25). The scoping reviews checked most of the items on the checklist, with three reviews (22, 24, 25) scoring 17.5 out of 20 items. Gmitroski et al. (23) checked 17 items, followed by Ebuenyi et al. (21) with 15. A higher score shows that the scoping review followed Cooper's et al. (19) checklist more closely, with stronger methods and clearer reporting, while a lower score points to more gaps in quality and reporting. Details about the quality appraisal of the scoping reviews can be found in Figure 2.

#### 3.3.3 Study overlap corrected covered area (CCA)

To check the level of study overlapping, we followed the CCA method proposed by Pieper et al. (26). In our included reviews (C = 40), we had a total of 931 (*N* = 931) studies, out of which 582 (R = 582) were unique, resulting in a slight overlap (1.53%). The complete overlapping table can be found in Supplementary Material 4. This low rate of overlapping suggests largely different sets of primary studies. This reduces redundancy and means that the evidence base covers a broader set of studies.

### 3.4 Factors associated with employment for people with SMI

To address our first research questions— “What are the psychological factors associated with employment status for individuals with severe mental illness (SMI)?” and “What are the positive vs. negative correlates of employment?”—we identified eight evidence-based factors, categorized as facilitators and barriers. The reviews included in this overview provided stronger evidence for social and clinical factors, which we present together for a more comprehensive understanding. Our synthesis also highlighted sociodemographic characteristics as relevant correlates of employment among individuals with SMI, partially addressing the question: “Do these associations vary based on education level or social functioning?”.

With regard to diagnostic subgroups, we could only partially answer the question: “Do these associations vary based on diagnosis type?”. Most reviews focused on individuals with psychotic disorders, although two studies (27, 28) reported that some supported employment interventions may be less effective for people with depression compared with those with psychotic disorders.

A large share of the included reviews examined interventions for occupational rehabilitation, primarily supported employment models. Thus, the first two facilitators—supported employment and other vocational or psychological interventions—address the question: “Does enrollment in rehabilitation programs facilitate employment for individuals with a psychiatric diagnosis?”.

Finally, the three barriers we identified were all linked to hospitalization and the illness trajectory. These findings address the question: “Do prior hospitalizations act as obstacles or moderators in gaining employment for psychiatric patients?” Evidence suggests that repeated hospitalizations can significantly hinder employment prospects in this population. In what follows, each of the identified aspects is detailed.

#### 3.4.1 Facilitators

Supported employment interventions (SE). Supported employment models aim to offer individualized support for people with disabilities to gain and maintain employment in the competitive labor market. Individual Placement and Support (IPS) is the most prominent SE model, designed primarily for individuals with SMI to facilitate rapid entry into the workforce. Out of the 40 included reviews, 19 focused on supported employment or interventions derived from it, such as IPS (3, 27–43). One review (34) reported no advantage of SE or IPS over treatment-as-usual (TAU), particularly in the short term (12 months). However, Crowther et al. (34) found significant long-term effects at 24 months [OR = 0.92, 95% CI (0.85, 0.99)] and 36 months [OR = 0.88, 95% CI (0.82, 0.96)], suggesting SE may outperform TAU over time. Three scoping reviews (21, 23, 25) also provided supportive evidence, showing SE and IPS improve employment outcomes for both obtaining and maintaining work (23, 25), as well as facilitating return-to-work (21). For example, IPS increased the odds of employment more than 14-fold compared with controls (23). Table 1 provides a detailed view of studies that approach interventions and their efficacy.

All reviews examined populations with SMI, with five focusing exclusively on psychotic disorders (30, 35, 39, 40, 43). Evidence on diagnostic subgroups was mixed: while Brinchmann et al. (32) suggested that results may generalize across diagnostic categories, Hellstrom et al. (27) reported no added benefit of IPS over TAU for individuals with depressive symptoms. Similarly, de Winter (28) and Campbell et al. (33) concluded that IPS is highly effective overall, but its effects are stronger for people with psychotic disorders.

Regional differences were also explored. Brinchmann et al. (32) and Killaspy et al. (36) found no significant regional variation in IPS effectiveness. Brinchmann et al. (32) noted a marginal effect mediated by legal protections, yet IPS consistently outperformed TAU across countries. In contrast, Bond et al. (29) reported stronger effects in U.S.-based studies compared with non-U.S. settings.

Other interventions. Six additional interventions were identified across nine reviews. Most showed evidence of being effective for gaining, maintaining, or returning to work, with the exception of prevocational training. This approach was examined in one review (34), which found no evidence that it was more effective than standard care. Other interventions included vocational rehabilitation (44, 45), cognitive remediation (46, 47), occupational training/occupational therapy (22, 48, 49, 63), and the Clubhouse Model (50, 51).

Vocational rehabilitation: Sengupta et al. (44) reported employment rates of 60% over 18 months. They found no significant differences between participants with and without alcohol use disorder [41.2% vs. 39.3%, χ^2^(1) = 0.046, *p* = 0.83] or drug use disorder [33.3% vs. 43.5%, χ^2^(1) = 1.35, *p* = 0.25]. Michon et al. (45) examined psychiatric vocational rehabilitation in patients with psychotic disorders, with employment rates ranging from 22% to 53%.

Cognitive remediation: Chan et al. (46) found that participants with psychotic disorders who received computer-assisted cognitive remediation (CACR) had higher employment rates compared to controls (41% vs. 24%), and worked on average 19.5 days longer [95% CI (2.5–36.6) days]. Traditional cognitive remediation (47) showed a significant effect on employment outcomes [SMD = 0.41, 95% CI (0.10, 0.72)] and employment duration [SMD = 0.48, 95% CI (0.30, 0.67)]. Abidin et al. (52) reviewed seven studies where cognitive remediation was typically a complementary intervention to IPS or other vocational services. While no study found direct employment improvements, CR enhanced psychosocial functioning.

Occupational training/therapy: Kirsh et al. (48) found that combining occupational training with treatment-as-usual significantly improved depressive symptoms [−2.8, 95% CI (−5.5, −0.2)], increased remission [+18%, 95% CI (7%, 30%)], and boosted long-term return-to-work in good health [+24%, 95% CI (12%, 36%)]. At three-month follow-up, 27.2% of participants were employed, and 16.3% were in training or education. The reviews included individuals with schizophrenia, PTSD, psychosis with comorbidities such as depression, phobias, or chronic pain. Edgelow et al. (22) concluded that for individuals with stress disorders (e.g., PTSD), person-centered occupational training delivered by interdisciplinary teams enhanced return-to-work outcomes (63). Arbesman et al. (49) also highlighted activity-based interventions (e.g., household tasks), particularly when paired with motivational interviewing and multidisciplinary collaboration.

Clubhouse Model: Battin et al. (50) and McKay et al. (51) evaluated this model across SMI populations. Battin et al. (50) found it especially effective for schizophrenia: 67.1% of Clubhouse members gained employment vs. 38.6% of users of other psychiatric rehabilitation services [OR = 3.24, 95% CI (1.6, 6.6)]. For other diagnoses, rates were lower but still significant [58.7% vs. 41.8%, OR = 1.9, 95% CI (1.25, 2.9)]. However, some comparisons with assertive community treatment (PACT) showed mixed results, with CH members less likely to achieve job placement [60% vs. 74%, OR = 0.53, 95% CI (0.26, 1.06)]. McKay et al. (51) reported no significant differences between Clubhouse and other interventions overall, but at six-month follow-up, a significantly higher proportion of Clubhouse members were employed (24% vs. 2%, *p* < 0.01).

Sociodemographic characteristics. Tse et al. (53) found that three sociodemographic variables (higher education, married, younger age) were associated with successful employment outcomes (the weighted ES ranged from 0.18 to 0.23 – small to moderate – and the average correlation was 0.21). Michon et al. (45) bring supportive evidence regarding marital status (married). Other reviews state that higher education represents a facilitator for employability of the psychiatric population; however, no statistical data were available: Bouwmans et al. (54) – younger age, more education (31); – younger age (45); – higher education (55); – marital status (56); – being in a relationship (25); - male gender (54); – barrier – later age of illness, facilitator – education.

Stability and reduced severity of mental illness. Ebuenyi et al. (21) found that stable and reduced symptom severity contribute to job retention, stability making employment retention two times more likely [OR = 2.19, 95% CI (1.27, 3.78)] and low symptom severity five times [OR = 4.86, 95% CI (1.51, 15.63)] more likely for young adults with a mental health diagnosis. De Winter et al. (28) analyzed the relationship between employment outcomes and lower symptoms at baseline and discovered a favorable effect [χ^2^(1) = 20.48; *p* < 0.01].

#### 3.4.2 Barriers associated with employment for people with SMI

Negative symptoms. One meta-analysis (53) found that greater negative symptoms were associated with poorer employment outcomes, although the effect sizes were small to moderate. Similarly, Michon et al. (45) reported that less severe symptoms were linked to higher employment rates. A scoping review by Ebuenyi et al. (21) also found that in four out of eight included studies, the presence and severity of illness were self-reported impediments to employment.

Across other reviews, symptom severity consistently emerged as a barrier. For instance, a longitudinal study included in Gilbert et al. (56) found that each additional week spent in a major depressive episode reduced the likelihood of employment by 5%. Tsang et al. (57) similarly classified the severity of illness as a barrier to employment in schizophrenia, with mixed findings: it was reported in two studies and confirmed as a barrier in one, while psychiatric symptomatology was cited as a barrier in ten studies, though six reported it as a nonsignificant predictor. Neuropathology was identified as a significant predictor in two studies out of four examined.

Additional reviews identified psychiatric and negative symptoms as barriers to employment (38, 40, 45, 54, 55), although without detailed data. Negative symptoms also act as barriers to maintaining or returning to work when present as comorbidities (58). For example, Glozier et al. (2002), cited in (58) found that the onset of psychotic symptoms after stroke reduced the likelihood of employment [OR = 0.42, 95% CI (0.22, 0.80)]. Likewise, Jorge et al. (2005), cited in (58) reported that the onset of mood disorders following traumatic brain injury predicted poorer vocational outcomes (Wald χ^2^ = 4.9, *p* = 0.03).

Multiple hospitalizations and a long course of illness. Having more hospitalizations has been consistently identified as a significant negative factor for vocational outcomes. The weighted effect sizes (ES) of variables related to hospitalization ranged from 0.17 to 0.31, indicating small effects. Similarly, the course of illness was negatively associated with employment outcomes, with an average weighted ES of 0.29 (53). Tsang et al. (57) and Michon et al. (45) also classified hospitalization duration as a significant negative predictor of employment, while Bouwmans et al. (54) highlighted a continuous illness course as a barrier. Nevertheless, findings are not entirely consistent. Kinoshita et al. (37) reported evidence from two RCTs that did not support an association between employment and either higher or lower rates of hospital admissions [RR = 0.71, 95% CI (0.53, 0.96)].

Treatment intensity. One meta-analysis found that treatment intensity or implication of multiple specialists in the development of employment strategies for persons with mental disorders can act as barriers to employment (combining cognitive remediation and psychiatric rehabilitation for 3.2 h of cognitive remediation per week for 33 sessions – B = −0.74; *p* < 0.01) (47).

#### 3.4.3 Other potential factors associated with employment

Many of the included reviews (*n* = 16) present factors associated with gaining or maintaining employment, as well as barriers to employment, in a narrative way without consistently reporting statistical data. These insights remain relevant to our research questions. The factors are detailed in Table 3 and may expand the conceptual framework of employment for people with SMI beyond narrowly defined clinical, interventional, or demographic predictors. By considering both evidence-based findings and narrative reports, the analysis becomes more integrative.

Among the identified factors, the most frequently reported barriers were negative or general psychiatric symptoms, mentioned in five reviews (38, 40, 45, 55, 57), with depressive symptoms specifically highlighted (27, 40). Substance abuse was identified in three sources (27, 28, 57), while the use of neuroleptic drugs was also noted as a barrier (43, 57).

Additional impediments were linked to labor market factors, such as recruitment into designated jobs for individuals with SMI (51), lack of workplace adjustments, or difficulties in the job search process (38). Socio-demographic characteristics were also described as barriers: unemployment history (38, 40, 59), low levels of education (56), and limited work experience (38).

## 4 Discussion

This overview of reviews identified seven categories of evidence-based factors – supported employment interventions, vocational rehabilitation, cognitive remediation, occupational training, Clubhouse Model, sociodemographic characteristics, and stability and reduced severity of symptoms- associated with employment for people with SMI, therefore fully addressing our first research question, “What are the psychological factors associated with employment status for individuals with severe mental health issues?”. Our findings revealed four facilitators. The first two refer to interventions: the first one, (1) participating in SE interventions, describes structured, individualized support for competitive employment, and the second one, (2) other interventions, describes enhancement of work readiness and functional skills. The third facilitator, (3) sociodemographic characteristics, reflects personal attributes associated with better vocational outcomes. The last facilitator, (4) stability and reduced severity of mental illness, indicates improved symptom management and functional consistency. Further, the results discussed three barriers. The first one, (i) negative symptoms, refers to the existence of clinical features that impede functionality, and the next one, (ii) multiple hospitalizations and a long course of illness, represents instability and cumulative functional decline. The last barrier, (iii) treatment intensity, suggests instances that may reduce availability of capacity for work activities. The most researched factor is represented by the SE interventions, specifically IPS (3, 29, 37). Other interventions included vocational rehabilitation, cognitive remediation, occupational training, and the Clubhouse model (22, 46, 50). Besides, our findings highlight several other potential factors associated with employment among individuals with SMI. Sociodemographic characteristics—such as higher education, younger age, and marital status—were positively linked to employment outcomes (45, 53). In contrast, certain clinical characteristics, including neuropsychological deficits, psychiatric comorbidities beyond the primary diagnosis, and negative symptoms, were consistently described as barriers (40, 43, 55). Taken together, these findings indicate that while evidence-based vocational interventions can substantially enhance employment outcomes, their success might be influenced by underlying psychological and clinical variables. Therefore, the research question regarding whether enrollment in rehabilitation programs facilitates employment can be answered affirmatively. Across the included reviews, participation in structured vocational and psychosocial rehabilitation programs consistently emerged as one of the strongest facilitators of employment outcomes for individuals with psychiatric diagnoses.

Our results align with a recent umbrella review by Patmisari et al. (12), which highlighted the superiority of supported employment (SE) models, particularly IPS, over other types of interventions for gaining, maintaining, and returning to work. The present umbrella review extends this understanding of SE models, though we note an overlap of 60.7% with the studies included in Patmisari et al. (12) (CCA: R = 28, *N* = 45, C = 2). The detailed overlap file is provided in Supplementary Material 5. Consistent with our findings on cross-country differences in SE effectiveness, Patmisari et al. (12) also reported stronger outcomes in U.S. studies. While we identified legal protection (32) as a potential moderator of the IPS–employment link, Patmisari et al. (12) pointed to GDP growth as another possible explanatory factor. Importantly, there are clear variations in the relationship between psychological and employment factors depending on diagnosis type. SE interventions appear less effective for individuals with depressive symptoms, substance use disorders, and anxiety (12, 27, 28). By contrast, for schizophrenia and bipolar disorder, SE interventions demonstrate significantly stronger effects (27, 35), with no substantial differences between the two diagnoses, contrary to Holm et al. (4). Hence, while many diagnoses are not approached, our research question regarding the variations in relationships based on the type of diagnosis is only partially answered, with most of the literature focusing almost exclusively on psychotic disorders.

Three reviews found cognitive remediation to be a positive predictor of employment (46, 47). However, this finding cannot be generalized to the entire psychiatric population, as most participants in these reviews had schizophrenia or schizoaffective disorder (97% in (46) or were on the psychotic spectrum [>75% in (47)]. Although both reviews were rated “critically low”, they each met four out of seven AMSTAR critical domains and conducted a risk of bias assessment. Still, they did not account for risk of bias when interpreting results, nor did they follow a pre-registered protocol or conduct a sufficiently comprehensive literature search. Thus, these findings should be interpreted with caution. Other vocational interventions identified in this review include vocational training, psychiatric rehabilitation, and the Clubhouse model (45, 48, 51), which may also be effective. Similar to Patmisari et al. (12), our findings highlight the need for further research on these interventions, as current evidence remains scattered.

Consistent with prior literature, several individual and social factors were identified as facilitators of employment. Reduced symptom severity and illness stability were associated with greater job retention (21, 28), while higher education, younger age, and supportive relationships were linked to more favorable employment outcomes (45, 53). Thus, there are variations in employment outcomes based on education level and social functioning. Individuals with higher educational attainment and stronger social skills demonstrated greater employability and job stability, likely due to enhanced cognitive resources, adaptive coping, and access to supportive networks (45, 53, 57). Conversely, lower education and poorer social functioning were associated with reduced work participation and increased risk of long-term unemployment (54, 56). However, these findings were largely derived from narrative data and low-quality reviews, so the evidence should be interpreted cautiously – hence our research question concerning the education level and social functioning is only partially addressed. These findings align with evidence highlighting the psychological and social benefits of employment, which extend beyond economic participation to improvements in autonomy, self-sufficiency, and social inclusion (1, 3). Jäckel et al. (5) also reported significant associations between hospitalizations and employment outcomes in people with SMI, a finding supported by four additional reviews included in this study. Furthermore, Guedes de Pinho et al. (2) identified social support as a key factor in employment among psychiatric populations, an aspect our analysis did not directly emphasize.

More negative symptoms and greater illness severity were consistently identified as barriers to employment for people with SMI, both through statistical evidence (45, 53) and narrative accounts (57). Luciano and Meara (7) examined the relationship between mental illness severity and employment outcomes, finding that employment rates declined with increasing symptom severity. While individuals without symptoms reported an employment rate of 75.9%, those with severe symptoms had substantially lower rates (54.5%). In addition, prior hospital commitments were found to act as hinderers rather than moderators of employment outcomes. Repeated psychiatric hospitalizations and a long course of illness reduce the likelihood of gaining or maintaining employment (45, 53, 54). The weighted effect sizes for hospitalization-related variables ranged between 0.17 and 0.31, indicating a small negative effect (53). These findings answer our research question, “Do previous hospital commitments act as hinderers or moderators for gaining employment for patients with a psychiatric diagnosis?”. They suggest that frequent hospital admissions disrupt vocational trajectories, reflecting the cumulative impact of illness severity, functional decline, and social withdrawal. Only limited evidence (37) suggested no clear association, implying that contextual factors such as treatment continuity and post-discharge support may moderate this relationship. Notably, findings on supported employment (SE) interventions indicate that these programs yield better outcomes for diagnoses typically associated with greater illness severity, such as schizophrenia and other psychotic disorders (28). This suggests that symptom severity may be a more meaningful determinant of vocational success than diagnostic category alone, underscoring the importance of SE programs in mitigating the vocational impact of severe symptom profiles.

Another important dimension emerging from this synthesis is the impact of repeated hospitalizations and a prolonged illness course on employment outcomes. Tse et al. (53) and Michon et al. (45) found that frequent psychiatric hospitalizations not only reflect illness severity but also disrupt vocational pathways. The weighted effect sizes indicate a small negative effect, underscoring the importance of clinical stability as a key factor for sustained employment. Similarly, Bouwmans et al. (54) highlighted that a continuous illness trajectory further diminishes opportunities by impairing social functioning and vocational identity. Treatment intensity also emerged as a relevant factor. van Duin et al. (47) observed that adding intensive cognitive remediation to psychiatric rehabilitation may, under certain conditions, overwhelm patients, thereby hindering rather than facilitating employment. These findings raise critical questions about intervention design: although comprehensive support is essential, excessive treatment demands may deplete time and energy needed for vocational participation. Thus, tailoring treatment intensity to individual needs is crucial to ensure that therapeutic programs remain enabling rather than burdensome. Hence. the second research question, “What are the negative correlates of employment and the positive ones?” was substantially answered, but mostly in a highly descriptive manner due to the evidence being moderate to low. Most of the reviews had significant methodological limitations.

Our findings suggest that there is a vast diversity of factors that might facilitate or hinder the employment of people with SMI. While there is consistent evidence in support of interventions, especially the ones following the SE model, none of them consider theoretical frameworks from work and organizational psychology that take into account workplace dynamics, motivation, leadership, or organisational culture and climate. This gap reflects a broader issue in workplace mental health research, which continues to suffer from a lack of integration across disciplines such as clinical psychology, social work, and organisational behavior (64). Consequently, our understanding of employment in the psychiatric population remains fragmented, overlooking the complex and contextual factors that shape employment experiences and outcomes for this specific group. Integrating such frameworks could offer a more comprehensive understanding of how contextual and systemic factors within workplaces influence the success and sustainability of supported employment outcomes. Moreover, greater theoretical integration would allow researchers to explore how diverse experiences of mental illness, intersecting identities, and cultural contexts shape inclusion and recovery within the workplace. Therefore, we recommend future research to explore how the identified psychological, social, and contextual factors are associated with employment, also through other theoretical models, such as the Sustainable Career Model (60, 61). We recommend the Sustainable Career Model, as it is an emerging model that approaches the development of a career in a dynamic way by balancing the personal resources and environmental demands over time. Unlike traditional rehabilitation perspectives, the Sustainable Career Model considers how individual resources interact with contextual factors such as leadership, organizational culture, and work design over time. This could make it relevant for people with SMI, whose employment outcomes depend not only on access to work but also on ongoing support, workplace adaptation, and recovery (60, 61). By emphasizing the dynamic interplay between health, happiness, and productivity, the model could provide a more integrative framework for examining how inclusive organizational practices and supportive environments can foster both employability and well-being. Through the model, research could bridge vocational rehabilitation and organizational psychology, promoting a more integrated and sustainable approach to inclusion in competitive employment.

### 4.1 Limitations

Although our findings contribute to outlining a potential theoretical framework for the employment of individuals with SMI, the review also has several limitations. The main limitation concerns the methodological quality of the included reviews, which was generally low. Most were rated as critically low on AMSTAR-2 due to shortcomings in protocol registration, risk of bias assessment, and consideration of publication bias. While the included scoping reviews (21–25) met most of the quality appraisal criteria, their findings still require corroboration through systematic reviews and meta-analyses of higher methodological rigor. This is particularly important because many factors were reported narratively rather than supported by consistent statistical data, limiting the ability to estimate effect sizes. Additionally, most reviews focused predominantly on psychotic disorders (43, 52, 54, 57), leaving other diagnostic categories, such as mood or personality disorders, comparatively underexplored. Therefore, we recommend that future research prioritize high-quality, preregistered systematic reviews that specifically address underrepresented diagnostic groups.

### 4.2 Future research

Our findings suggest that there is a vast diversity of factors that might facilitate or hinder the employment of people with SMI. While there is consistent evidence in support of interventions, especially the ones following the SE model, none of them consider theoretical frameworks from work and organizational psychology that take into account workplace dynamics, motivation, leadership, or organisational culture and climate. This gap reflects a broader issue in workplace mental health research, which continues to suffer from a lack of integration across disciplines such as clinical psychology, social work, and organisational behavior (64). Consequently, our understanding of employment in the psychiatric population remains fragmented, overlooking the complex and contextual factors that shape employment experiences and outcomes for this specific group. Integrating such frameworks could offer a more comprehensive understanding of how contextual and systemic factors within workplaces influence the success and sustainability of supported employment outcomes. Moreover, greater theoretical integration would allow researchers to explore how diverse experiences of mental illness, intersecting identities, and cultural contexts shape inclusion and recovery within the workplace. Therefore, we recommend future research to explore how the identified psychological, social, and contextual factors are associated with employment, also through other theoretical models, such as the Sustainable Career Model (60, 61). We recommend the Sustainable Career Model, as it is an emerging model that approaches the development of a career in a dynamic way by balancing the personal resources and environmental demands over time. Unlike traditional rehabilitation perspectives, the Sustainable Career Model considers how individual resources interact with contextual factors such as leadership, organizational culture, and work design over time. This could make it relevant for people with SMI, whose employment outcomes depend not only on access to work but also on ongoing support, workplace adaptation, and recovery (60, 61). By emphasizing the dynamic interplay between health, happiness, and productivity, the model could provide a more integrative framework for examining how inclusive organizational practices and supportive environments can foster both employability and well-being. Through the model, research could bridge vocational rehabilitation and organizational psychology, promoting a more integrated and sustainable approach to inclusion in competitive employment. Therefore, we propose two research questions for future research to encourage the inclusion of vocational rehabilitative principles in the general organizational culture: “How can supported employment programs be adapted to better integrate principles of organizational psychology?” and “How do personal resources interact with environmental demands to predict sustainable employment trajectories in individuals with SMI?”.

### 4.3 Conclusions

Employment outcomes among people with severe mental illness or a psychiatric diagnosis are shaped by a range of factors. Our review identified seven such factors—four facilitators and three barriers. Participating in supported employment, engaging in other vocational interventions, advantageous sociodemographic characteristics and stability with recuced symptom severity emerged as facilitators. The strongest evidence supports the inclusion of individuals with SMI in supported employment programs, particularly Individual Placement and Support (IPS). These interventions significantly increase opportunities for gaining, maintaining, and regaining employment through individualized plans for integration into the competitive labor market. Other interventions, such as cognitive remediation and occupational training, also show promise, although the supporting evidence is less comprehensive. Conversely, barriers such as negative symptoms, repeated hospitalizations with a prolonged illness course, and treatment intensity can impair functioning and limit vocational success. Despite these insights, the majority of included reviews were rated as critically low on AMSTAR-2, primarily due to the lack of risk-of-bias assessments. The only exception was four scoping reviews, which received high-quality appraisals. Given these methodological limitations, further high-quality research is needed.

## Data Availability

The original contributions presented in the study are included in the article/Supplementary Material, further inquiries can be directed to the corresponding author.

## Supplementary material

Supplementary Material 1 Preferred reporting items for overviews of reviews.

Supplementary Material 2 Search strategy.

Supplementary Material 3 List of excluded studies.

Supplementary Material 4 Study overlap.

Supplementary Material 5 Study overlap with Patmisari et al. (2024).
