# Supplementary material for: Barriers and facilitators of employment in severe mental illness: an umbrella review
Source: Front Rehabil Sci. 2026 Jan 22;6:1731096. doi: 10.3389/fresc.2025.1731096 (PMC12872853; doi:10.3389/fresc.2025.1731096)
Supplement: Supplementary Material 2 — Search strategy. [file Table2.docx]

**ONLINE SUPPLEMENTARY MATERIAL 2, FOR "BARRIERS AND FACILITATORS OF EMPLOYMENT IN SEVERE MENTAL ILLNESS: AN UMBRELLA REVIEW”**

*Search strategy*

| Search: (((((cognitive functioning[Title/Abstract] OR social support[Title/Abstract] OR employment[Title/Abstract] OR unemployment[Title/Abstract] OR employed[Title/Abstract] OR unemployed[Title/Abstract] OR job[Title/Abstract] OR work[Title/Abstract] OR workplace[Title/Abstract] OR occupation[Title/Abstract] OR vocational[Title/Abstract] OR hire[Title/Abstract] OR labor[Title/Abstract]) AND (psychiatric[Title/Abstract] OR "mental illness"[Title/Abstract] OR "mental disorder"[Title/Abstract] OR "mental disorders"[Title/Abstract] OR "mental health"[Title/Abstract] OR "mentally ill"[Title/Abstract] OR "mental illness"[Title/Abstract] OR schizophrenia[Title/Abstract] OR psychosis[Title/Abstract] OR bipolar[Title/Abstract] OR "personality disorders"[Title/Abstract] OR "mood disorders"[Title/Abstract] OR "affective disorders"[Title/Abstract] OR PTSD[Title/Abstract] OR "dissociative disorder"[Title/Abstract] OR depression[Title/Abstract] OR depressive[Title/Abstract] OR anxiety[Title/Abstract])) AND (diagnosis[Title/Abstract] OR diagnostic[Title/Abstract] OR diagnose[Title/Abstract])) NOT (review[Title/Abstract] OR overview[Title/Abstract] OR meta-analysis[Title/Abstract]))) AND (social skills[Title/Abstract] OR "work performance"[Title/Abstract] OR "social functioning"[Title/Abstract] OR "self-efficacy"[Title/Abstract] OR "work history"[Title/Abstract] OR "community functioning"[Title/Abstract] OR "autonomy"[Title/Abstract] OR "personal agency"[Title/Abstract]) Filters: Abstract |
| --- |
