# Supplementary material for: Barriers and facilitators of employment in severe mental illness: an umbrella review
Source: Front Rehabil Sci. 2026 Jan 22;6:1731096. doi: 10.3389/fresc.2025.1731096 (PMC12872853; doi:10.3389/fresc.2025.1731096)
Supplement: Supplementary Material 5 — Study overlap with Patmisari et al. (2024). [file Table5.docx]

**ONLINE SUPPLEMENTARY MATERIAL 5, FOR “BARRIERS AND FACILITATORS OF EMPLOYMENT IN SEVERE MENTAL ILLNESS: AN UMBRELLA REVIEW”**

*Study overlap between current review and Patmisari et al. (2024)*

| Study ID | Review 1 | Review 2 | Overlap |
| --- | --- | --- | --- |
| Abidin et al. | 1 | 1 | 1 |
| Arbesman et al. | 1 | 0 | 0 |
| Bond et al. (2012) | 1 | 1 | 1 |
| Bond et al. (2014) | 1 | 0 | 0 |
| Bond et al. (2022) | 1 | 1 | 1 |
| Brinchmann et al. | 1 | 0 | 0 |
| Campbell et al. | 1 | 1 | 1 |
| Crowther et al. | 1 | 1 | 1 |
| de Winter et al. | 1 | 0 | 0 |
| Heffernan et al. | 1 | 1 | 1 |
| Hellstrom et al. | 1 | 0 | 0 |
| Killaspy et al. | 1 | 0 | 0 |
| Kinoshita et al. | 1 | 1 | 1 |
| Modini et al. | 1 | 1 | 1 |
| Noyes et al. | 1 | 0 | 0 |
| Richter et al. | 1 | 0 | 0 |
| Rinaldi et al. | 1 | 0 | 0 |
| Smith et al. | 1 | 0 | 0 |
| Thompson et al. | 1 | 0 | 0 |
| Twamley et al. | 1 | 1 | 1 |
| Charette-Dussault et al. 2019 | 0 | 1 | 0 |
| Chen et al. 2020 | 0 | 1 | 0 |
| Moen et al. 2021 | 0 | 1 | 0 |
| Kinn et al. 2021 | 0 | 1 | 0 |
| Johanson et al. 2023 | 0 | 1 | 0 |
| Wallstroem et al. 2021 | 0 | 1 | 0 |
| Charzyńska et al. 2015 | 0 | 1 | 0 |
| Aguey-Zinsou et al. 2022 | 0 | 1 | 0 |
| Frederick and VanderWeele 2019 | 0 | 1 | 0 |
| Carmona et al. 2017 | 0 | 1 | 0 |
| Dewa et al. 2018 | 0 | 1 | 0 |
| Drake et al. 2012 | 0 | 1 | 0 |
| Mallick et al. 2022 | 0 | 1 | 0 |
| Suijkerbuijk et al. 2017 | 0 | 1 | 0 |
| Bond et al. 2008 | 0 | 1 | 0 |
| Crowther et al. 2001 | 0 | 1 | 0 |

Review 1 - current review; Review 2 - Patmisari et al. (2024)
